# Involvement of the Arg^566^ residue of *Aeromonas sobria* serine protease in substrate specificity

**DOI:** 10.1371/journal.pone.0186392

**Published:** 2017-10-12

**Authors:** Hidetomo Kobayashi, Tadamune Otsubo, Fumiteru Teraoka, Kiyoshi Ikeda, Soshi Seike, Eizo Takahashi, Keinosuke Okamoto, Toru Yoshida, Hideaki Tsuge, Hiroyasu Yamanaka

**Affiliations:** 1 Laboratory of Molecular Microbiological Science, Faculty of Pharmaceutical Sciences, Hiroshima International University, Hiroshima, Japan; 2 Laboratory of Synthetic Organic Chemistry, Faculty of Pharmaceutical Sciences, Hiroshima International University, Hiroshima, Japan; 3 Collaborative Research Center of Okayama University for Infectious Diseases in India, National Institute of Cholera Enteric Diseases JICA Building ID Hospital Campus, Kolkata, India; 4 Department of Bioresource and Environmental Sciences, Faculty of Life Sciences, Kyoto Sangyo University, Kyoto, Japan; United States Army Medical Research Institute of Infectious Diseases, UNITED STATES

## Abstract

*Aeromonas sobria* serine protease (ASP) is an extracellular serine protease secreted by the organism. Here, we identified the amino acid residue of ASP that contributes to substrate specificity by using both synthetic peptides and biological protein components. The results showed that the arginine residue at position 566 (Arg-566) of ASP, which is located in the extra occluding region of ASP close to an entrance of the catalytic cavity, is involved in the substrate specificity. A substitutional point mutation of the Arg-566 residue of ASP to Ala residue (ASP[R566A]) caused a decrease of the proteolytic efficiency for a certain substrate. In addition, ASP lost the ability to recognize the primary substrate by such a point mutation, and ASP[R566A] reacted to a wide range of synthetic substrates. It is likely that Arg-566 causes an interaction with the amino acid residue at position P3 of the substrate, which is the third amino acid residue upstream from the cleavage site. Another study using ORF2 protein, a chaperone protein of ASP, further suggested that Arg-566 could also play an important role in interaction with ORF2. We therefore conclude that the Arg-566 residue of ASP is likely responsible for the selection of substrates.

## Introduction

*Aeromonas* species are Gram-negative facultative anaerobic organisms that are ubiquitous in various aquatic environments such as fresh and brackish water areas [1]. More than ten *Aeromonas* species have been reported, but the number of *Aeromonas* species involved in human diseases is more limited. Two species in particular, *A*. *sobria* and *A*. *hydrophila*, are reported to be strongly associated with human diseases [2–5]. The main symptom caused by infection with either of these organisms is gastroenteritis [1,6], but an infected patient's condition sometimes progresses to a more serious state, and severe acute extra-intestinal diseases such as sepsis, phlegmon and myonecrosis can be triggered by the infection [7], with a higher probability of such triggering in immunocompromised patients.

Virulent strains of *Aeromonas* produce a variety of extracellular toxins including hemolysin, cytotonic enterotoxin, heat-stable enterotoxin, heat-labile enterotoxin, lipase, and several types of proteases [8–13]. These toxins might increase the virulence of *Aeromonas*. For example, *Aeromonas sobria* serine protease (ASP) (EC 3.4.21.121) destroys not only structural and functional proteins but also the proteins essential for host defense [14,15]. It is therefore likely that ASP is involved in the onset of extra-intestinal diseases such as sepsis, phlegmon and myonecrosis.

Our previous studies demonstrated that nascent ASP synthesized from the DNA requires the chaperone protein to become the mature ASP (i.e., the active form of ASP) [16]. The chaperone protein is encoded just downstream of *asp*. Therefore, the chaperone protein has been designated as an ORF2 (open reading frame 2) [16]. In our PSI-BLAST search using the ORF2 sequence, we found that operons consisting of genes homologous to *asp* and *orf2* are present in four genera other than *Aeromonas*: *Vibrio*, *Shewanella*, *Chromobacterium*, and *Pseudoalteromonas* [17]. However, it is not yet known how the chaperone protein works on a specific protein at the molecular level. Our recent analysis of the complex of ASP with ORF2 showed that the carboxyl terminal region of ORF2 tightly interacts with the amino acid residues around the catalytic site of ASP [17]. It seems that the interaction prevents the nascent ASP from the proteolysis by itself (autolysis) during the folding process.

**Fig 1:**
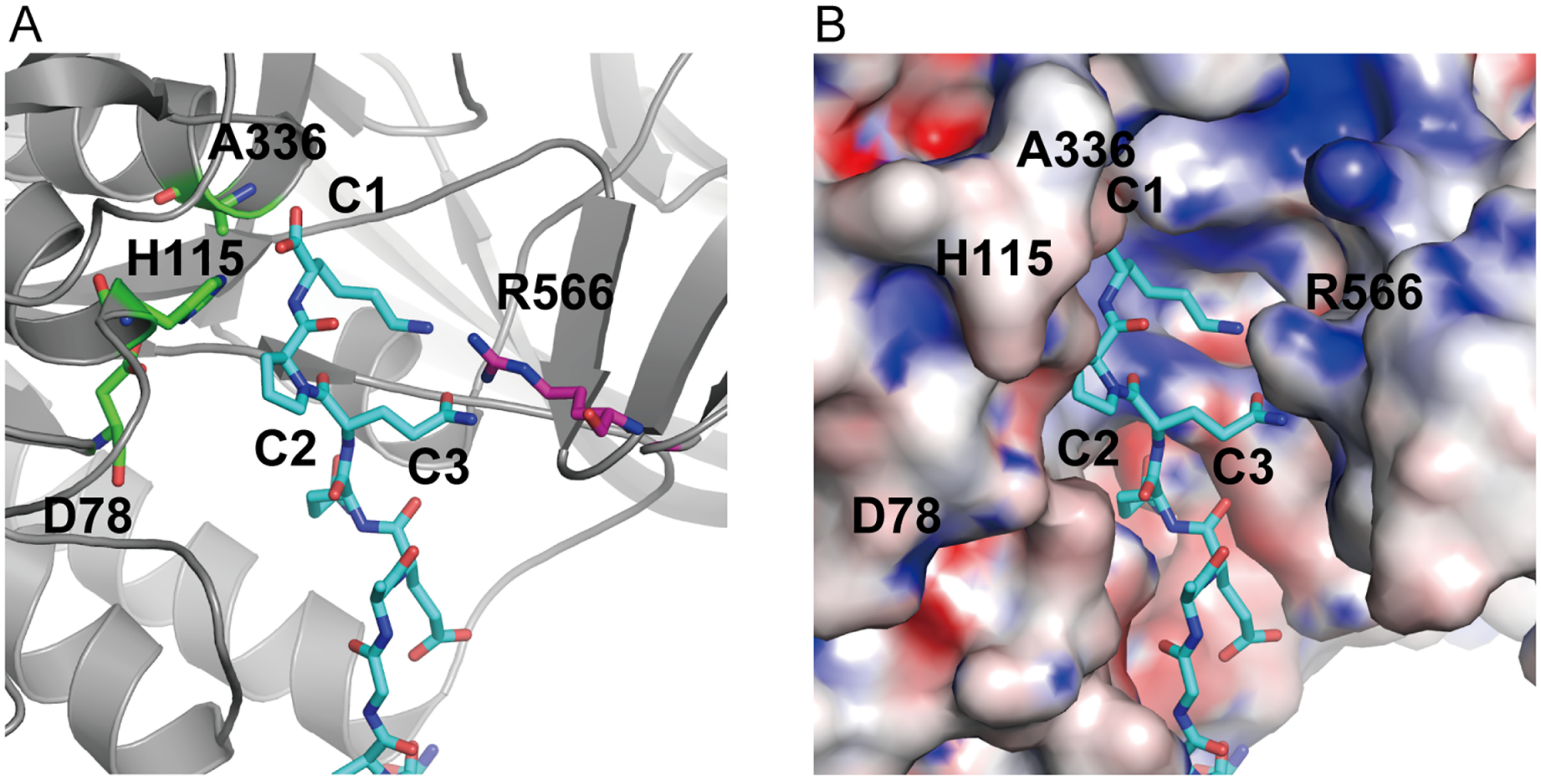
Close-up view of the interaction site of ASP(S336A) with ORF2. (A) To investigate the interaction of ASP with ORF2, we incubated the inactive mutant ASP, ASP(S336A), with ORF2. After incubation with ORF2, the generated complex of ASP(S336A) with ORF2 was purified and its complex structure was analyzed [17]. The structures of ASP and ORF2 are depicted by a ribbon model and a stick model, respectively. The positions of the three amino acid residues of ORF2 (C1; Lys, C2; Pro, and C3; Gln residues which are the first, second and third amino acid residues from the carboxyl terminal end of the ORF2, respectively) and those of the four amino acid residues of ASP(S336A) (D78, H115, A336 and R566) are shown. D78, H115 and A336 are amino acid residues constituting the catalytic triad (in wild-type ASP, the amino acid residue at position 336 is S). (B) Electrostatic potential surface map of the catalytic region of the complex of ASP(S336A) with ORF2. The location of ORF2 is indicated by stick model. The properties of several amino acid residues shown in the figure are described in the legend for panel A. Negatively and positively charged residues are shown in red and blue, respectively.

The carboxyl terminal amino acid residues at positions C1, C2 and C3 (the first, second, and third amino acids from the carboxyl terminal end) of ORF2 are Lys, Pro and Gln. As shown in Fig 1A, the carboxyl terminal Lys residue of ORF2 seems to interact with the catalytic center residue at position 336 of ASP. Further examination showed that the Gln residue at position C3 of ORF2 is close to the Arg-566 residue of ASP (Fig 1A). The Arg-566 residue of ASP is one of the amino acid residues comprising the extra occluding region of ASP, and the position of Arg-566 in the stereo structure is close to the catalytic site of ASP [17,18]. From these observations, we speculate that Arg-566 residue may have a role to interact with external molecules such as the chaperone protein ORF2. If such function will be exerted in the interaction of ASP with its substrate molecules, we assume that the Arg-566 residue of ASP will also play an important role in the proteolytic action with the substrates.

To test this possibility, we first conducted experiments using synthetic substrates. Since our previous research revealed that ASP preferentially hydrolyzes the synthetic substrate whose amino acid residues at positions P1 and P2 (i.e., the first and second amino acid residues upstream from the cleavage site, respectively) are Lys residues [19], in the present study we synthesized several substrates composed of three amino acid residues whose amino acid residue at the P3 position varied but amino acid residues at both P1 and P2 positions were Lys residues. We then examined the ability of ASP to cleave these substrates, in order to determine how the reactivity of ASP to the substrate is changed by the difference in the P3 residue. We also created mutant ASP in which the Arg-566 residue was substituted with an alanine residue, and we then examined the ability of the mutant ASP to cleave these substrates.

Our previous studies revealed that ASP induces vascular leakage, reduces blood pressure through an activation of the kallikrein/kinin system [14], and causes the formation of pus and edema through the action of anaphylatoxin C5a [14]. Thus, since ASP works as a major pathogenic factor in *A*. *sobria* infection, it is also important to examine the effect of ASP against these biological components. We therefore further investigated the efficiency of the proteolytic actions of both ASP and the mutant ASP against biological protein components such as fibrinogen and high-molecular-weight kininogen as the substrates.

The results obtained in our investigation indicate that the Arg-566 of ASP contributes to the selection of the substrate of the protease. It is likely that these effects are due to differences in the recognition ability of the amino acid structure at the P3 position of the substrate by the Arg-566 of ASP.

## Materials and methods

### Bacterial strains and plasmids

An atypical strain of *Aeromonas sobria* T94 which does not produce ASP was used as a host strain. To produce a large amount of ASP, we transformed *A*. *sobria* T94 with the plasmid pSA19-5528 containing both *asp* and *orf2*, as described [16].

### Site-directed mutagenesis

To substitute the arginine residue at position 566 (Arg-566) of ASP to the alanine residue, we site-directly mutated *asp* on pSA19-5528 according to the polymerase chain reaction (PCR)-based one-step mutagenesis method [20,21]. The PCR was carried out using KOD plus high-fidelity DNA polymerase (Toyobo, Osaka, Japan). The primers 5′-ctctctgctcaatgccgagacccgtgag-3′ and 5′- ctcacgggtctcggcattgagcagagag-3′ were used for mutation of the target gene. The mutation was verified by DNA sequencing. The plasmid thus obtained was designated pSA19-5528[R566A].

### Purification of ASP

ASP was purified as described [16]. Briefly, *A*. *sobria* T94 transformed with either pSA19-5528 or pSA19-5528[R566A] was cultured in Luria-Bertani broth with shaking (160 rpm) at 37°C for 16 hr. After cultivation, the bacterial cells were removed from the culture fluid by centrifugation, and ammonium sulfate was added to the culture supernatant until 60% saturation was reached. After the solution was kept at 4°C for 15 hr, the precipitate yielded was collected by centrifugation (18,000 × *g* for 45 min). The precipitate collected was then dissolved in 5 ml of 1 mM sodium phosphate buffer (pH 7.4) and dialyzed against the same buffer.

This obtained crude ASP preparation was loaded onto a hydroxyapatite column (Bio-Rad Laboratories, Hercules, CA) equilibrated with 1 mM sodium phosphate buffer (pH 7.4). After the column was washed with the same buffer, the adsorbed material was eluted with a linear gradient of 1 to 120 mM sodium phosphate buffer (pH 7.4). Fractions showing the proteolytic activity were collected and concentrated by ultrafiltration (Vivascience, Hannover, Germany). Thus partially purified sample was further separated by a Superdex 200 column (GE Healthcare, Milwaukee, WI) in a high-performance liquid chromatography (HPLC) system, and the fractions containing ASP were collected. The solution obtained was used as the purified ASP preparation. The purity of ASP in the preparation was confirmed by SDS-PAGE.

### Preparation of the various fluorogenic peptide substrates

Fmoc-Lys(Boc)-MCA (1.18 g) [22] was dissolved in 20% piperidine-DMF (15 ml) and stirred for 30 min at room temperature. The mixture was dried under reduced pressure to give H-Lys(Boc)-MCA, and the residue yielded (433 mg) was dissolved in DMF/CH_2_Cl_2_ (2:1, 20 ml). Then, Fmoc-Lys(Boc)-OH (1.06 g, 2.25 mmol), HOBT (305 mg, 2.25 mmol) and WSC·HCl (433 mg, 2.25 mmol) were added to the solution. The resulting mixture was stirred for 16 hr at room temperature. The reaction mixture was diluted with AcOEt, and the organic layer was washed successively with HCl (1 mol/l, two times), water (two times) and brine (one time). The organic layer obtained was dried over Na_2_SO_4_, filtered and concentrated under reduced pressure. The residue obtained was purified by silica gel column chromatography (n-hexane:AcOEt = 2:3 to 1:2 as an eluent) to give the desired Fmoc-Lys(Boc)-Lys(Boc)-MCA (911 mg, 71% yield).

**Fig 2:**
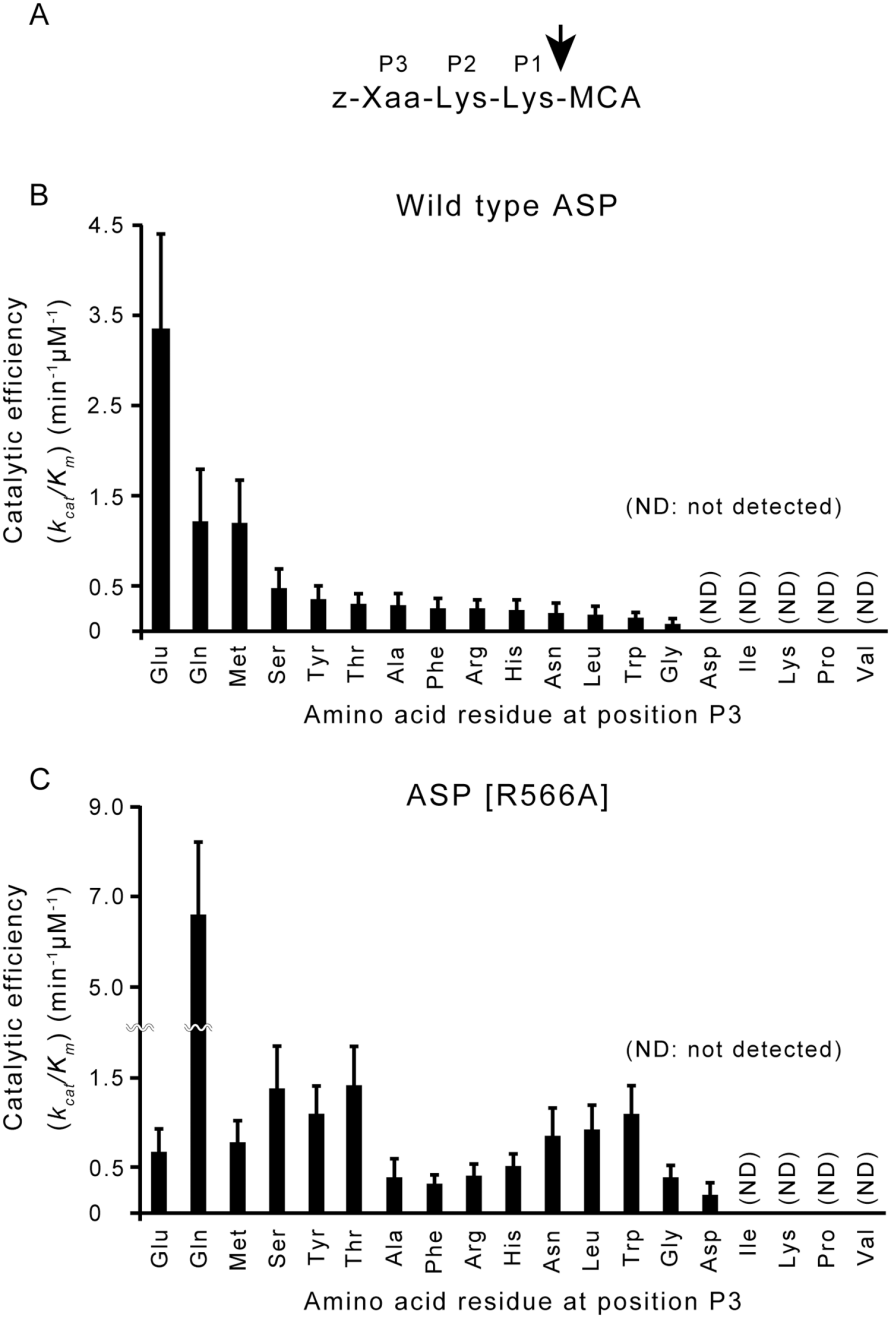
Cleavage of various fluorogenic peptide substrates by wild-type ASP and the mutant ASP. (A) Schematic diagram of fluorogenic peptide substrates synthesized in this study. The arrow indicates the position where ASP cleaves [19]. The amino acid residue at P3 (shown as Xaa in the figure) is different in each substrate. Z indicates a benzyloxycaronyl group. (B), (C) *k*_cat_/*K*_m_ values calculated from the reaction of wild-type ASP (B) and ASP(R566A) (C) with the various substrates. The reaction was performed as described in the Materials and Methods section, and the results obtained are shown in Table 2. The numerical values shown in Table 2 are illustrated here.

Z-X-Lys(Boc)-Lys(Boc)-MCA (Z, benzyloxycabonyl; X, amino acid residue): A solution of Fmoc-Lys(Boc)-Lys(Boc)-MCA (300 mg, 351 μmol) in 20% piperidin-DMF (5 ml) was stirred for 30 min to give H-Lys(Boc)-Lys(Boc)-MCA. The solution was dried under reduced pressure, and the residue yielded was dissolved in DMF solution (4 ml). The solution (400 μl) was mixed with acid-labile protected Z-X-OH (100 μmol), and then HOBT (1 mol/l in DMF:CH_2_Cl_2_ = 2:1, 100 μl) and WSC·HCl (1 mol/l in DMF:CH_2_Cl_2_ = 2:1, 100 μl) were successively added to the mixture. The resulting mixture was stirred for 21 hr. The reaction mixture was diluted with AcOEt, and the organic layer was washed successively with water (two times), diluted NaHCO_3_ solution (one time) and brine (one time), dried over Na_2_SO_4_, filtered and concentrated under reduced pressure. The obtained residue was dissolved in 50% TFA-CH_2_Cl_2_ (2 ml), stirred for 1 hr and concentrated under reduced pressure. This synthesized crude fluorogenic substrate was purified by a preparative HPLC system (column: YMC-Actus Triart C18 250 × 20 mm I.D.; eluent: A = H_2_O, B = CH_3_CN, C = CH_3_CN containing 1% TFA A:B:C = 80:10:10 (0 min) − 80:10:10 (5 min) − 60:10:30 (35 min), flow rate 4 ml/min). The desired substrate (whose sequence is shown in Fig 2A) was obtained as TFA salt. The products were confirmed by the mass spectrum. The high-resolution mass spectra (HRMS) were measured on AccuTOF (JMS-T100LC) equipped with an electrospray ion source (JEOL Ltd. Tokyo, Japan). Mass spectrometry data and the yield for each compound were shown in Table 1.

**Table 1:** High-resolution mass spectrometry data and the yield for synthetic substrates.

No.	Substrates	obtained	HRMS	molecular weight	
		(mg)	formula for [M+H]+	calculated	observed
1	Z -EKK-MCA	9.8	C_35_H_47_N_6_O_9_	695.34045	695.34104
2	Z -QKK-MCA	7.6	C_35_H_48_N_7_O_8_	694.35643	694.35701
3	Z -MKK-MCA	13.1	C_35_H_49_N_6_O_7_S	697.33834	697.33377
4	Z -SKK-MCA	7.6	C_33_H_45_N_6_O_8_	653.32989	653.32581
5	Z -YKK-MCA	7.2	C_41_H_50_N_7_O_7_	729.36119	729.36302
6	Z -TKK-MCA	10.2	C_34_H_47_N_6_O_8_	667.34554	667.3449
7	Z -AKK-MCA	9.7	C_33_H_45_N_6_O_7_	637.33497	637.3344
8	Z -FKK-MCA	13.5	C_39_H_48_N_6_O_7_	713.36627	713.3663
9	Z -RKK-MCA	9.7	C_36_H_52_N_9_O_7_	722.39897	722.40012
10	Z -HKK-MCA	4.9	C_36_H_47_N_8_O_7_	703.35677	703.35682
11	Z -NKK-MCA	7.7	C_34_H_46_N_7_O_8_	680.34078	680.34045
12	Z -LKK-MCA	11.4	C_36_H_51_N_6_O_7_	679.38192	694.38234
13	Z -WKK-MCA	10.1	C_35_H_49_N_6_O_7_	752.37717	752.37704
14	Z -GKK-MCA	8.9	C_32_H_43_N_6_O_7_	623.31932	623.31813
15	Z -DKK-MCA	7.8	C_34_H_45_N_6_O_9_	681.3248	681.32506
16	Z -IKK-MCA	16.8	C_36_H_51_N_6_O_7_	679.38192	679.38222
17	Z -KKK-MCA	13.4	C_36_H_52_N_7_O_7_	694.39282	694.39175
18	Z -PKK-MCA	9.5	C_35_H_47_N_6_O_7_	663.35062	663.35388
19	Z -VKK-MCA	12.1	C_34_H_47_N_6_O_8_	665.36627	665.36639

### Measurement of the proteolytic activity of ASP or ASP[R566A] against synthetic peptide substrates

Each fluorogenic substrate was dissolved in dimethylformamide at a concentration of 10 mM. Since our previous studies demonstrated that ASP shows sufficient proteolytic action in phosphate buffer solution (pH ranging from 7.2 to 7.5) [17–19], in the present study we mixed 2 μl of the substrate solution (10 mM) with 148 μl of ASP or ASP[R566A] (1 nM) in 20 mM sodium phosphate buffer (pH 7.4) in order to examine the cleavage of each substrate by ASP or ASP[R566A]. The mixture was incubated at 37°C for 30 min. After incubation, the reaction was stopped by adding 150 μl of acetic acid.

As the substrate releases 7-amino-4-methyl-coumarin by receiving the cleavage at the P1 site (the bond following Lys-Lys), we determined the proteolytic activity of ASP or ASP[R566A] by measuring the fluorescence generated. That is, the fluorescence of the solution was measured with at *λ*_ex_ = 340 nm and *λ*_em_ = 440 nm. A spectrofluorometer (ARVO MX 1420 Multilabel Counter, Perkin Elmer, San Jose, CA, USA) was used. Estimations of maximum velocity (*V*_max_) and the Michaelis-Menten constant (*K*_m_) were performed by using nonlinear regression analysis within GraphPad Prism software (GraphPad Software Inc., La Jolla, CA).

### Cleavage of various proteins by ASP and by ASP[R566A]

We reported that ASP induces vascular leakage and reduces blood pressure through an activation of the kallikrein/kinin system [14,15]. Here we examined the proteolytic action of ASP and that of ASP[R566A] against biological components such as fibrinogen (Sigma-Aldrich, St Louis, MO) and high-molecular-weight kininogen (Enzyme Research Laboratories, South Bend, IN). We incubated fibrinogen or high-molecular-weight kininogen solution (200 nM) with 0.4 nM of ASP or ASP[R566A] in 20 mM phosphate buffer (pH 7.4) at 37°C. After various periods of time, 15 μL of the mixture was withdrawn, followed by the addition of 1.5 μL of phenylmethylsulfonyl fluoride (PMSF) (10 mM) to terminate the reaction. The reaction mixtures obtained were analyzed by sodium dodecyl sulfate-polyacrylamide gel electrophoresis (SDS-PAGE) under reducing conditions using a 12% polyacrylamide gel. Oriole fluorescent gel stain (Bio-Rad Laboratories, Hercules, CA) was used for protein staining. The experiments were done triplicate.

### Analysis of the inhibitory action of ORF2 against ASP or ASP[R566A]

The inhibitory action of ORF2 against ASP or ASP[R566A] was analyzed as previously described [17]. Briefly, to determine the IC_50_ of ORF2 for ASP or ASP[R566A], the activity of ASP or ASP[R566A] (20 nM) in the presence of various amount of purified ORF2 (0 to 200 nM) was measured using a synthetic substrate, Boc-Glu-Lys-Lys-MCA (66 μM). The IC_50_ was defined by calculating the ORF2 concentration at which 50% of the activity of ASP or ASP[R566A] was blocked. IC_50_ was determined using GraphPad Prism software (GraphPad Software Inc., La Jolla, CA). This experiment was performed in triplicate, and the data was shown as the average value. To compare the inhibitory strength of ORF2 against ASP with that against ASP[R566A], we further observed the degradation of ORF2 in the solution containing various amount of ASP or ASP[R566A]. Briefly, 60 μM of purified ORF2 was incubated for 5 min at 37°C with various concentrations (0–10 μM) of ASP or ASP[R566A] in 20 mM phosphate buffer (pH 7.4). After the incubation, the reaction was terminated by the addition of PMSF, and each reaction mixture was analyzed by SDS-PAGE under reducing conditions using an 18% polyacrylamide gel. This experiment was done triplicate.

### Molecular modeling

The structure models of ASP was carried out using the PyMOL analytical system (http://www.pymol.org) [23].

## Results and discussion

### Cleavage of synthetic substrate by wild-type ASP

In our previous work, we analyzed the degradation products from the fluorogenic synthetic substrates subjected to the action of ASP by high-performance liquid chromatography and mass spectrometry [19], and the results demonstrated that ASP cleaves the carboxyl terminal side of the synthetic substrate where P1 and P2 are Lys residues. In the present study, we synthesized several substrates linked to a fluorogenic compound, MCA, whose amino acid residues at positions P1 and P2 were Lys residues but the P3 residue varies. Since it is thought that the fluorogenic compound MCA is specifically released from synthetic substrates subjected to the action of ASP [19], we speculated that the enzyme activity of ASP can be analyzed by measuring the fluorescence intensity of MCA released in the reaction mixture.

Then each synthetic substrate was reacted with ASP, and the values of *K*_m_, *V*_max_, and *k*_cat_ were calculated from the results obtained. These values are shown in Table 2. In order to clarify the strength of the proteolytic activity of ASP against each substrate, we calculated the *k*_cat_/*K*_m_ value, which reflects the catalytic efficiency of ASP against the substrate. That is, when ASP exerts its proteolytic action more efficiently with a certain substrate, the value of the *k*_cat_/*K*_m_ becomes larger.

**Table 2:** Kinetic constants in the hydrolysis of synthetic substrates by wild-type ASP and the mutant ASP[R566A].

No.	Substrates	Wild-type ASP ^a^			ASP [R566A] ^a^		
		*K*_*m*_ (μM)	*k*_*cat*_ (min^-1^)	*k*_*cat*_/*K*_*m*_ (min^-1^μM^-1^)	*K*_*m*_ (μM)	*k*_*cat*_ (min^-1^)	*k*_*cat*_/*K*_*m*_ (min^-1^μM^-1^)
1	Z -EKK-MCA	8.92±2.54	29.81±2.71	3.34±1.07	15.08±2.79	10.26±0.71	0.68±0.25
2	Z -QKK-MCA	34.86±9.87	42.15±5.83	1.21±0.59	3.63±0.81	23.98±1.29	6.60±1.60
3	Z -MKK-MCA	18.43±2.79	22.07±1.33	1.20±0.48	6.84±2.72	5.40±0.63	0.79±0.23
4	Z -SKK-MCA	29.35±7.98	13.93±1.75	0.47±0.22	11.15±2.03	15.30±0.95	1.37±0.47
5	Z -YKK-MCA	16.23±5.82	5.85±0.81	0.36±0.14	5.89±1.24	6.43±0.38	1.09±0.31
6	Z -TKK-MCA	22.03±8.80	6.55±1.10	0.30±0.13	8.22±1.56	11.61±0.68	1.41±0.44
7	Z -AKK-MCA	34.40±15.51	10.14±2.23	0.29±0.14	40.55±12.81	15.97±2.59	0.39±0.20
8	Z -FKK-MCA	23.42±8.70	5.98±0.95	0.26±0.11	10.67±4.97	3.38±0.53	0.32±0.11
9	Z -RKK-MCA	16.07±5.54	4.05±0.53	0.25±0.10	8.11±3.49	3.37±0.45	0.42±0.13
10	Z -HKK-MCA	27.16±8.29	6.40±0.88	0.24±0.11	4.67±1.64	2.42±0.22	0.52±0.14
11	Z -NKK-MCA	70.00±31.60	13.73±3.78	0.20±0.12	13.76±2.33	11.70±0.72	0.85±0.31
12	Z -LKK-MCA	43.61±24.92	8.06±2.42	0.18±0.10	6.84±3.08	6.36±0.84	0.93±0.27
13	Z -WKK-MCA	18.39±5.38	2.74±0.32	0.15±0.06	5.89±1.24	6.43±0.38	1.09±0.31
14	Z -GKK-MCA	52.24±78.04	4.50±3.75	0.09±0.05	13.36±5.27	5.16±0.73	0.39±0.14
15	Z -DKK-MCA	ND^b^			91.15±77.76	18.54±10.43	0.20±0.13
16	Z -IKK-MCA	ND^b^			ND^b^		
17	Z -KKK-MCA	ND^b^			ND^b^		
18	Z -PKK-MCA	ND^b^			ND^b^		
19	Z -VKK-MCA	ND^b^			ND^b^		

^a^ Wild-type ASP and ASP [R566A] were used at the same concentration in this experiment.

^b^ND, not detected.

The *k*_cat_/*K*_m_ values obtained from each reaction are shown in Table 2 and illustrated in Fig 2B. As shown in the figure, the *k*_cat_/*K*_m_ value of wild-type ASP was the largest in the reaction with the substrate of which the P3 residue was Glu. The figure also shows that the *k*_cat_/*K*_m_ ability of wild-type ASP to cleave the substrate possessing Gln at P3 was moderate, but the cleavage of the substrate by ASP definitely occurred. Interestingly, the ability of wild-type ASP to cleave other substrates (except the substrate possessing Met at P3) was very weak (Fig 2B).

Our previous study of the molecular structure of ASP indicated that the extra occluding region of ASP is located close to its catalytic cavity. The structural analysis also revealed that the Arg-566 residue seems to be adjacent to the P3 residue when a substrate enters the catalytic cavity of ASP [17,18]. We therefore suspect that the Arg-566 residue plays an important role in the selection of a substrate by recognizing the structure of the side chain at P3. Our present findings showed that the substrate possessing Glu at P3 is most sensitive to the cleavage action by ASP, and that the substrate possessing Gln at P3 is the next sensitive (Table 2, Fig 2B). The side chain structures of these two amino acids resemble each other. Thus, the results obtained in this experiment support our hypothesis that Arg-566 of ASP contributes to the substrate specificity of ASP by offering a suitable space for the amino acid residue of a substrate at P3. To test our hypothesis, we examined the effect of a point mutation at the Arg-566 residue of ASP on the proteolytic action against the same series of synthetic substrates.

### Cleavage of synthetic substrate by mutant ASP of which the Arg-566 residue is substituted with an alanine residue

We site-directly mutated *asp* to substitute Arg-566 with Ala-566. Then we purified the mutant ASP (ASP [R566A]) and analyzed the enzymatic reaction of the mutant ASP against various synthetic substrates. We measured the values of *K*_*m*_, *V*_max_, *k*_cat_ and *k*_cat_/*K*_m_ of each reaction using the same method as that performed in the analysis of the reaction of wild-type ASP with synthetic substrates. The values obtained are listed in Table 2, and the *k*_cat_/*K*_m_ values are illustrated in Fig 2C.

The results showed that the efficacy of ASP to cleave these substrates is largely changed by the mutation. It was clear that several substrates possessing Gln, Ser, Tyr, Thr, Asn, Leu, and Trp at position P3 were efficiently cleaved by the mutant ASP. These results indicate that ASP lost the ability to recognize the primary substrate by the point mutation of Arg-566 to Ala-566. That is, the substitution of Arg-566 with Ala-566 of ASP caused a loss of the high substrate specificity of ASP, although the cleavage activity itself seemed to be unchanged by the mutation (Fig 2C). Notably, compared to the data of *K*_m_/*k*_cat_ values shown in Table 2, the efficacy of ASP to cleave the substrate possessing Glu at P3 was markedly decreased by the mutation. In contrast, the mutant ASP[R566A] showed high degradation activity against the substrates that had Gln at P3. These changes can be considered from the analysis of kinetic parameters as follows.

Comparing kinetic parameters of ASP for the tripeptide Z-EKK-MCA with that for Z-QKK-MCA, the *k*_cat_ value of ASP for Z-QKK-MCA was 1.4 times higher than that for Z-EKK-MCA but the *K*_m_ value of ASP for Z-QKK-MCA was 3.9-fold higher than that for Z-EKK-MCA, meaning that ASP has higher affinity with Z-EKK-MCA than Z-QKK-MCA although proteolytic rate of ASP against Z-QKK-MCA is slightly higher than Z-EKK-MCA. Taken comprehensively, it seems that ASP can more efficiently hydrolyze Z-EKK-MCA than Z-QKK-MCA. Thus, it is thought that the tripeptide with Gln as the third residue from the carboxyl terminus is the poorer substrate for ASP.

In contrast, comparing the kinetic parameters of ASP[R566A] for the tripeptide Z-EKK-MCA with that for Z-QKK-MCA, the *k*_cat_ value of ASP[R566A] for Z-QKK-MCA was higher than that for Z-EKK-MCA. In addition to this result, the *K*_m_ value of ASP[R566A] for EKK-MCA was 4.15-fold higher than that for Z-QKK-MCA. These results indicates that the higher preference of the mutated enzyme (ASP[R566A]) for substrate Z-QKK-MCA over Z-EKK-MCA is due to both a higher catalytic constant and a lower *K*_m_ value.

Furthermore, we compared the catalytic constants of ASP and ASP[R566A] for the tripeptides either Z-EKK-MCA or Z-QKK-MCA. For both substrates, the turnover is higher for the wild-type enzyme. Even with Z-QKK-MCA, which is the preferred substrate for the mutant enzyme and the not-so-preferred substrate for the wild-type enzyme, the *k*_cat_ value of ASP was still higher than that of the mutant ASP. The biggest difference between the two enzymes is in the *K*_m_ value with Z-QKK-MCA as substrate. That is, the *K*_m_ value was almost 10 times lower for the mutant enzyme ASP[R566A], explaining why catalytic efficiency of the mutant enzyme against Z-QKK-MCA as reflected in the *k*_cat_/*K*_m_ value is so much higher as compared to the wild-type ASP, despite the lower *k*_cat_ value for the mutant enzyme.

In conclusion, above results strongly suggest that the substrates with Glu and Gln at P3 are the most favored substrates among all synthetic substrates examined in this study for the wild ASP and the mutant ASP[R566A], respectively. We therefore think that the Arg-566 residue of ASP plays a key role in the selection of substrate, probably due to recognition of the side-chain structure at P3 of the substrate. We speculate that the positively charged side chain of Arg-566 may function to attract substrates with Glu at P3 to the catalytic domain and that the substitution of Arg-566 for Ala may lower such characteristics and attract a variety of substrates because of small side-chain structure. Further molecular biological studies are required to test this speculation.

In the above-described experiments, we used an artificially synthesized peptide as the substrate. The primary substrate of ASP in the natural condition is protein. The size of the substrate used in this experiment is small compared to those of the primary substrates. A small substrate might be profitable for approaching the catalytic site of the enzyme, because regions other than the cleavage site of the substrate often obstruct the approach to the substrate of the reaction site of the enzyme. Such obstruction does not occur in peptide substrates.

To determine whether the Arg-566 residue is related to the proteolytic action of ASP against biological protein components, we next examined the proteolytic actions of both the wild-type and the mutant ASP against fibrinogen and high-molecular-weight kininogen, which are already known as the biological protein substrates for ASP [14,15].

### Cleavages of biological protein components by wild-type ASP and the mutant ASP

**Fig 3:**
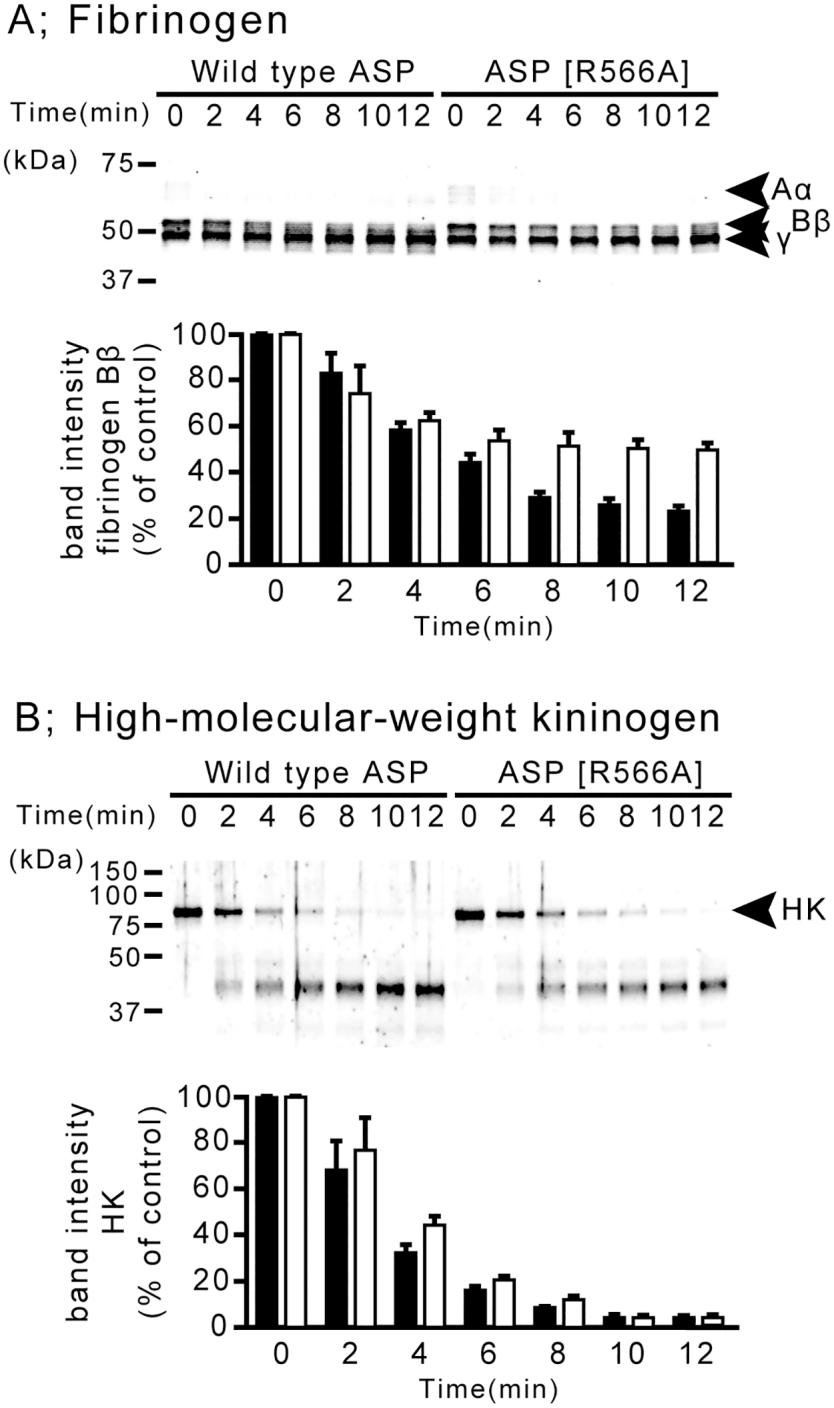
The cleavage of various proteins by ASP and ASP [R566A]. (A) Cleavage of fibrinogen by ASP and by ASP [R566A]. Human fibrinogen (200 nM) was incubated with ASP or ASP [R566A] (0.4 nM) for various periods of time (min). The locations of fibrinogen Aα, Bβ and γ chains are indicated by the arrows (upper panel). The band intensity of fibrinogen Bβ incubated with ASP (black bar) or ASP[R566A] (white bar) was also shown in lower panel. (B) Cleavage of high-molecular-weight kininogen by ASP or ASP [R566A]. High-molecular-weight kininogen (200 nM) was incubated with ASP or ASP [R566A] (0.4 nM) for various periods of time (min). The location of high-molecular-weight kininogen (HK) is indicated by the arrow (upper panel). The band intensity of HK incubated with ASP (black bar) or ASP[R566A] (white bar) was also shown in lower panel. All experiments shown in this figure were performed triplicate.

We observed the substrate cleavage by wild-type ASP and the mutant ASP, using fibrinogen and high-molecular-weight kininogen. Degradation of the components such as Aα and Bβ of fibrinogen by wild-type ASP occurred slightly faster than that by the mutant ASP (ASP[R566A]) (Fig 3A). Similarly, cleavage of high-molecular-weight kininogen (HK) by wild-type ASP proceeded faster than that by the mutant ASP (Fig 3B). These results mean that wild-type ASP hydrolyzes both fibrinogen and high-molecular-weight kininogen more efficiently than the mutant ASP. We therefore speculate that the Arg-566 residue of ASP also plays an important role in the proteolytic action against the biological protein substrates. However, this role seems to be limited because the decomposition reaction of the substrate was occurred even by the action of the mutant ASP. Further molecular biological analyses are required to clarify the role of the Arg-566 residue of ASP in the proteolytic action against these substrates.

### Inhibitory effect of ORF2 against the proteolytic activities of wild-type ASP and the mutant ASP

As described in the Introduction, nascent ASP synthesized from the DNA requires the chaperone protein, ORF2, to become the mature ASP (i.e., the active form of ASP) [16]. Based on our previous analysis of the structure of the complex of ASP-ORF2, we hypothesized that the carboxyl terminal region of ORF2 tightly interacts with the amino acid residues around the catalytic site of ASP [17]. It thus seems that ORF2 functions as a temporary inhibitor for ASP and supports the correct folding of ASP.

As indicated by the above-described results, it appears that the Arg-566 residue of ASP is involved in substrate selection. We therefore speculate that this residue may also be related to the interaction with the chaperone protein such as ORF2. To test this idea, we further examined the inhibitory effect of ORF2 against both wild-type ASP and the mutant ASP[R566A]. If the Arg-566 residue of ASP contributes to the interaction with ORF2, the inhibitory effect of ORF2 against ASP will be decreased by the point mutation of this residue. In contrast, since ORF2 (which is not engaged in the inhibitory action against ASP) will be gradually hydrolyzed by being subjected to the proteolytic action of ASP, it is expected that the degradation amount of ORF2 by the mutant ASP will be larger than that by wild-type ASP.

**Fig 4:**
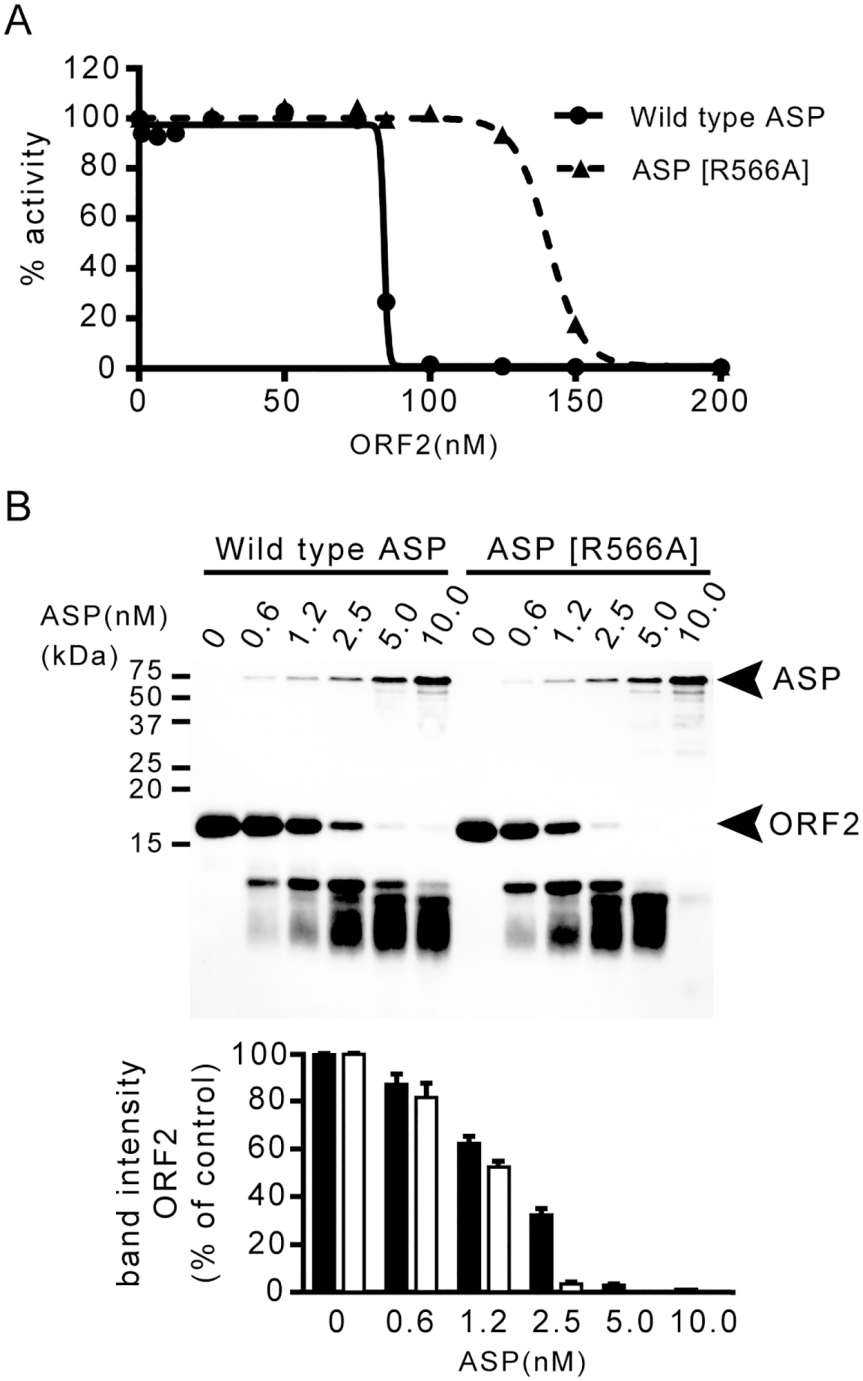
The inhibitory action of ORF2 against ASP or ASP[R566A] and the degradation of ORF2 by ASP or ASP[R566A] in the reaction mixture. (A) Determination of the IC_50_ for ORF2 against ASP or ASP[R566A]. ASP or ASP[R566A] activity against the synthetic substrate, Boc-Glu-Lys-Lys-MCA in the absence of ORF2 is defined as 100% activity. (B) Degradation of ORF2 by ASP or ASP [R566A]. A purified ORF2 preparation (60 μM) was incubated with various concentrations (0–10 nM) of ASP or ASP [R566A] solutions. The locations of intact ORF2 and ASP or ASP[R566A] are indicated by the arrows (upper panel). The band intensity of ORF2 incubated with ASP (black bar) or ASP[R566A] (white bar) was also shown in lower panel.

We first determined the concentration at which ORF2 inhibits the proteolytic activity of ASP or ASP[R566A] by 50% (IC_50_). As shown in Fig 4A, the result clearly indicated that ORF2 more strongly inhibited the proteolytic activity of wild-type ASP than that of ASP[R566A]. The IC_50_ values for ASP and ASP[R566A] were calculated as 84.3 nM and 140.6 nM, respectively. This result indicates that the inhibitory effect of ORF2 against ASP was decreased by the point mutation of the Arg-566 residue of ASP. We therefore suspect that the Arg-566 residue of ASP is also engaged in the interaction with ORF2.

Next, we observed the degradation of ORF2 in the solution containing various amount of ASP (0–10μM) mixed with ORF2 (60 μM). As shown in Fig 4B, the amount of ORF2 degraded within 5 min by action of wild-type ASP was less than that by reaction with the mutant ASP. For example, almost all of the ORF2 was degraded by incubation with 2.5 nM ASP[R566A] but not by incubation with 2.5 nM wild-type ASP. This result supports the validity of our hypothesis as described above. That is, it seems that ORF2 more strongly inhibits the proteolytic activity of ASP than that of ASP[R566A] probably due to efficient interaction with ASP and therefore the amount of ORF2 degraded by ASP is also less than that by ASP[R566A].

Considering these results along with the maturation process of ASP, such an inhibitory interaction occurring between ORF2 and ASP is thought to be important to prevent the nascent ASP from the proteolysis by itself (autolysis) during the maturation process. It is likely that the region including the Arg-566 residue of ASP also provides an appropriate conformation to receive the action of the chaperone protein ORF2.

ASP has been reported to be a member of the group of subtilisin-like serine proteases. Not only bacterial proteases such as subtilisin Carlsberg (3.4.21.62) and thermitase (3.4.21.62) but also eukaryotic proteases such as Kex2 (3.4.21.61) and furin (3.4.21.75) are included in that group [24]. In a previous study, we purified ASP with the size of 65 kDa and revealed its crystal structure at 1.65 Å resolution [18]. Our structural analysis revealed that the structure of the catalytic domain of ASP resembles those of other bacterial subtilisins, and that ASP possesses another domain that is not maintained in other bacterial subtilisins [18].

It is also interesting that Kex2, which is a protease from yeast cells, maintains another domain. In fact, the overall structure of ASP resembles that of Kex2 [18]. We thus compared the structure of ASP with that of Kex2 in detail and found that ASP possesses the extra occluding region in the vicinity of the catalytic region, but such a region is not found in Kex2 [18]. Our further examination of the structure of ASP showed that side chains of several amino acid residues in the extra occluding region extend into the catalytic cavity of ASP [18]. The side chain of the Arg-566 residue of ASP, which is one of the amino acid residues in the extra occluding region, notably protrudes into the catalytic cavity. The structure of ASP was thus clarified, but the role of the extra occluding region of ASP remains unclear.

In the present study, we observed that the ability of ASP to recognize substrates selectively is lost by the mutation of ASP from Arg-566 to Ala-566 (Table 2, Fig 2B and 2C). These results indicate that Arg-566 of ASP plays an important role in the association of the primary substrate with ASP. This finding guides the idea that the main roles of the extra occluding region of ASP are to detect and bind with the specific substrate of ASP.

Our results indicate that Arg-566 of ASP plays an important role in the selection of a genuine substrate by offering a suitable space for the residue at P3 of the substrate. As arginine is a positively charged amino acid residue, Arg-566 might act to draw the substrates possessing a negatively charged amino acid residue. Such drawing activity of ASP might be exhibited especially against small-sized substrates. Actually, in the reaction of wild-type ASP, the peptide substrate possessing Glu at P3 was efficiently cleaved by ASP (Table 2, Fig 2B). The peculiar property given by Arg-566 might help the efficient cleavage; that is, the ability of Arg-566 to offer a suitable space and draw a negatively charged amino acid residue might increase the *k*_cat_/*K*_*m*_ value of ASP in the cleavage of the substrates possessing Glu at P3. As we discussed in our previous study, the S4 site of Kex2 containing the Glu-255 residue (which is thought to interact with the P4 residue of the substrate) is reportedly involved in the selection of basic amino acids at P4 of the substrate [18], but no amino acid corresponding to the Glu-residue is present in ASP [18]. Instead, it is likely that the extra occluding region including the Arg-566 residue which notably protrudes into the catalytic cavity functions in substrate selection by ASP, as seen in that by Kex2. In addition, in the thrombin-catalyzed activation of factor VIII, it was reported that P3-P3' residues flanking scissile bonds in the factor VIII modulate substrate was cleaved by thrombin [25]. This report may be another example indicating that the amino acid residues adjacent to the cleavage site of a substrate largely affect the proteolytic action. Taking our present findings into account together with these examples, we propose that the Arg-566 residue located in the extra occluding region of ASP is involved in substrate selection.

**Fig 5:**
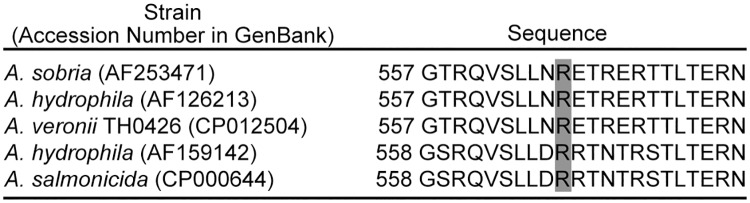
Sequence alignment around the position of 566 of several ASPs. The sequences shown are quoted from GenBank. The accession numbers of each sequence in GenBank are shown in the figure. The numbers at the left edge of the sequence indicate the position from the amino terminal end of each ASP. Arginine residues corresponding to Arg-566 of ASP of *A*. *sobria* 288 are indicated by shadowed characters.

As the DNA sequences of ASP and its homologous serine proteases were determined and their deduced amino acid sequences were published, we compared these deduced amino acid sequences around Arg-566 (Fig 5). The sequences around Arg-566 are well conserved in all proteases, supporting the idea that the region around Arg-566 is important for the action of ASP.

Extracellular proteases are often involved in the development of a bacterial infection by the activation of some inactive proproteins. The cleavage of proproteins must be done only at specific sites to generate active proteins. Therefore, the substrate specificity of the protease which is involved in the pathogenicity of such bacteria might be high. If the proproteins are decomposed into pieces by proteases, these features do not appear, because the decomposed fragments do not have such activity. For example, ASP is thought to be involved in the induction of vascular leakage and the reduction of blood pressure through the activation of the kallikrein/kinin system [14], in the promotion of human coagulation through the activation of prothrombin [26], and in the formation of pus and edema through the action of C5a [15]. We speculate that ASP acts as a virulence factor in the onset of these diseases through the site-specific cleavage of the proproteins that participate in the pathogenicity of *Aeromonas*. Further studies, especially *in vivo*, are necessary to demonstrate the virulence of ASP. Such studies could contribute to our understanding of the action of ASP as a pathogenic factor and to the development of novel inhibitory agents.
